# An agent-based secure privacy-preserving decentralized protocol for sharing and managing digital health passport information during crises

**DOI:** 10.7717/peerj-cs.1458

**Published:** 2023-07-18

**Authors:** Akram Y. Sarhan

**Affiliations:** Department of Information Technology, College of Computing and Information Technology, University of Jeddah, Jeddah, Saudia Arabia

**Keywords:** Privacy, Security, Multiagent systems, Digital health passports, Cryptography, Smart city, Pervasive computing, e-health, Blockchain, Privacy set intersection

## Abstract

The aim of this article is to identify a range of changes and challenges that present-day technologies often present to contemporary societies, particularly in the context of smart city logistics, especially during crises. For example, the long-term consequences of the COVID-19 pandemic, such as life losses, economic damages, and privacy and security violations, demonstrate the extent to which the existing designs and deployments of technological means are inadequate. The article proposes a privacy-preserving, decentralized, secure protocol to safeguard individual boundaries and supply governments and public health organizations with cost-effective information, particularly regarding vaccination. The contribution of this article is threefold: (i) conducting a systematic review of most of the privacy-preserving apps and their protocols created during pandemics, and we found that most apps pose security and privacy violations. (ii) Proposing an agent-based, decentralized private set intersection (PSI) protocol for securely sharing individual digital personal and health passport information. The proposed scheme is called secure mobile digital passport agent (SMDPA). (iii) Providing a simulation measurement of the proposed protocol to assess performance. The performance result proves that SMDPA is a practical solution and better than the proposed active data bundles using secure multi-party computation (ADB-SMC), as the average CPU load for SMDPA is approximately 775 milliseconds (ms) compared to about 900 ms for ADB-SMC.

## Introduction

The internet has made the world a small, global village, enabling people and businesses to interact and exchange ideas to solve various challenges on Earth. However, unpredictability, ambiguity, and complexity are significant issues of modern life in the 21st century (Hassankhani et al., 2021). For example, the death toll and economic damage due to unpredictable crises related to climate change and widespread diseases show how vulnerable humans are in the face of such calamities. Furthermore, the paucity of effective standardized international planning, policies, tools, strategies, and protocols to deal with sudden changes and disturbances (Hassankhani et al., 2021) makes it extremely difficult to interact adequately and efficiently with various phenomena. It, therefore, stands to reason to argue that, not only could innovative technology be a promising tool for addressing potential disasters, but also the need for efficient data and information management is essential—for example, SARS-CoV, H1N1, MERS-CoV, Ebola, Zika, and SARS-CoV-2 viruses.

The digitization of the healthcare process has also played a crucial role during crises in enhancing the healthcare systems *via* various emerging technologies, such as telemedicine, augmented reality, artificial intelligence, big data, electronic health records, and mobile health (Hassankhani et al., 2021). Moreover, the pandemic crisis of COVID-19 has accelerated the digitization of social life to the extent that e-learning, remote working, and remote services were all core tools in coping with the adversity (Van Wyk et al., 2020).

Besides data management and coordination, digital technology adoption is essential to the collection of data for better crisis management strategies. Many applications have been deployed for contact tracing, screening, health data information collection, symptom monitoring, facial recognition, global positioning system (GPS) data extractions, and facemask detection (Whitelaw et al., 2020; Elsayed et al., 2021). The integration of emerging technologies such as 5G wireless technology (Zhang et al., 2021), artificial intelligence (AI), blockchain, big data, drone (Al-Gburi et al., 2022), and cloud computing into crisis-based applications plays an indispensable role in handling crises, be it monitoring, preventing, or controlling. However, several issues and concerns have been raised, including the absence of robust interoperability, and the lack of global standardization on data collection between databases (Greene, McClintock & Durant, 2021), privacy, security (Borra, 2020), weak and insecure infrastructures (Raisaro et al., 2020), app storage, and implementation models.

The existence of global standards and interoperability between database institutions at the local or international level could enable intersectoral collaborations (Shokoohi, Osooli & Stranges, 2020) and support effective coordination and decision-making process at wide (Luengo-Oroz et al., 2020). However, the current technical limitations in interoperability and standardization, including privacy and security, restrict the scope of coordination between nations. Professor Ari Lightman from Carnegie Mellon stated, “As data becomes more of an asset, it becomes difficult to exchange that data across multiple different parties in an ecosystem” (Hern, 2021). Thus, app interoperability, including backend servers, must be essential for practical cross-border infection tracking and monitoring. However, there are some issues concerning whether to choose the centralized or decentralized model, the data sharing mechanisms, the mass of the public participant, the technical difficulties and functioning of the apps, and the reliability of mobile sensors and components, such as GPS, and Bluetooth signals (Ciucci & Gouardères, 2020).

Privacy-preserving is another critical matter that has raised serious concerns during the COVID-19 Pandemic. Mobile apps are essential in many nations to deal effectively with crises. However, such technologies have sparked privacy concerns about the mass information collection, the sharing, and the exposing of personal data with or without the consent of the user, as well as of the storing of such data in a centralized database or passing them to a trusted third-party server (TTP) (Wu, 2020; Borra, 2020). To cite an example, in the COVID-19 pandemic, there have been several concerns regarding the abuse of the contact tracing apps-based centralized model. Several individuals’ sensitive personal information and metadata have been collected, stored in a centralized database, and shared between local institutions. Furthermore, population movement has been tracked using several tools, such as credit card records, smartphone signals, CCTV footage, and mobile location data (Borra, 2020). Such collected information is vulnerable to data breaches, unwanted surveillance, and commercial advertisements (Sun et al., 2020).

Several countries introduced immunity passports to ease the lockdown policies and enable people to resume everyday life. The passport is a digital certificate granted to an individual to show that he/she is believed to have received complete vaccination, immunization, or some form of protection against the virus. However, despite the enormous benefits of such a digital health passport, several challenges have been raised concerning people’s civil liberties, including ethical and practical difficulties (Brown et al., 2020).

Although several articles have examined security and privacy features relating to crisis apps and digital health or immunity passports, to the best of my knowledge, there has not been a decentralized protocol for securely outsourcing sensitive data that uses agent-based technology as to provide the solutions, ideas, and features that are proposed in this article.

The motivation of this article, therefore, is to design a secure digital health passport protocol that has the characteristics and that serves the following purposes: To (i) perform anonymized data intersection among passengers’ digital health passports and local and international institutions while preserving complete privacy; (ii) ensure secure, shared information with full retention of user and apps data; (iii) propose a data retention policy that increases user trust and reduces privacy leakages and data storage cost; (iv) provide interoperable autonomous cross-border privacy-preserving digital solution to deal with cross-border international data protection standard; (v) minimize surveillance and provide anonymity for travelers during an interaction with cross-border agents, (vi) avoid having to register in any third party app and ensure free movement; and finally (vii) protect against abuse for discrimination (profiling), eliminate restrictions, and minimize economic damage. Please note that complete privacy in this article means a strong level of privacy that can ensure maximum anonymity. The private set intersection cardinality (PSI-CA) protocol does not reveal the data but shows the intersection size. Furthermore, we assume that the PSI-CA protocol does not leak any information concerning the data or the set intersection size.

Anonymizing historical data concerning visited countries should not cause harm because the authority can impose any role in their datasets. On the contrary, such action should present many benefits (i) avoids profiling or human error since the proposed smart passport manages movements based on the intersection results. Furthermore, (ii) avoids denying entry due to a stopover flight since most flights today include many connections. (iii) Avoid waiting in long lines for screening and monitoring vaccination status. Finally, (iv) presents an idea for digitizing people’s travel passports. However, the proposed protocol has limitations, as described in “Architecture and Design of the Proposed Scheme” and “SMDPA Simulation Experimentation”.

The article is structured into eight sections. Besides the first section of the Introduction, Section 2 is the relevant literature review. Section 3 presents a systematic review of crisis-based privacy-preserving apps, while Section 4 states the article’s core problem. Section 5 describes the architecture and design of the proposed scheme. Section 6 provides the simulation experimentation of the proposed solution, and Section 7 shows the results and discussion of the proposed work. Finally, Section 8 concludes the article, highlighting future directions of inquiry.

## Literature review

### Digital crisis management platforms and their privacy preserving

Digital crisis management platforms have the incredible potential to respond in a timely way during a crisis. MicroMappers (MM), for example, is a digital volunteer platform that uses AI for disaster response. Its associated tools for mining crisis-related information were submitted *via* volunteers and placed on the map. Google Crisis map (GCM) contains a U.S.-based set of layers concerning crises related to hazards, weather, response, and emergency preparedness.

Other tools and platforms created by Google for crisis management are Google Person Finder, Google Maps Engine Lite, Google Earth, and Google Public Alerts. However, such crowdsensing platforms must be integrated with encryption technology, as they are vulnerable to security threats and data leakage, insecure data dissemination, and system malfunction (Halder, 2017).

Digital crisis management mainly relies on smartphones, since they have expanded worldwide and altered how people live. Owing to their enormous utility and usefulness, they have become must-have tools, particularly in crisis-ridden times like ours. Furthermore, they have played an essential role in assisting authorities in crisis management.

Smartphones, nevertheless, are associated with many risks that have been an ongoing concern regarding these apps (Chan et al., 2020). Examples include collecting information without permission (Gnadinger, 2014); and extracting unneeded personal information through mobile app services and sometimes without users’ knowledge, jeopardizing users’ sensitive data and making it vulnerable to data leakage and hardware control (Zhu et al., 2016). Furthermore, insecure software apps have been criticized on account of several well-known cases presented as follows: (i) poor implementation (Fischer et al., 2017) and authorization, (ii) session management issues (Jain & Shanbhag, 2012), (iii) weak encryption, including the misuse of cryptography APIs and deployment model (Egele et al., 2013), and (iv) poor-skills software programmers.

#### Smartphone apps data privacy and security regulations

The development of smartphone apps to combat crises started in 2011 by Jon Crowcroft and Eiko Yoneki at Cambridge University (Borra, 2020). Several countries have proposed privacy, security, and data protection regulations and frameworks to govern, regulate and ensure compliance with how information is collected, maintained, used, and disseminated. Nevertheless, mobile app development has obstacles to bridging technical knowledge and privacy regulations. Such a lack of app privacy awareness for the user and developer has not facilitated the development process of privacy-based apps.

Yet, protecting the confidentiality of data during usage and dissemination continues to be a challenge. In addition, the massive data collection practice of mobile user data has raised serious concerns. Thus, several privacy-preserving digital data policies and regulations have been implemented to cope with data collection, storage, dissemination, and retention issues (Michael & Abbas, 2020; Hatamian, 2020).

#### Privacy-preserving apps deployment model

Privacy-preserving apps developed during COVID-19 have relied on centralized, decentralized, or hybrid models (Shubina et al., 2020). The centralized deployment model relies on trusted third-party servers (TTPs) for data processing, computation, and storing anonymous data and identities, including their cryptographic processes. Nonetheless, it is a bottleneck and a single point of failure, and is prone to several attacks, including side-channel and correlation attacks (Avitabile et al., 2020). In addition, its centralized storage databases are controlled by authorities.

Thus, several decentralized and multilevel security protocols have been proposed to tackle this issue (Sarhan & Carr, 2017; Sarhan, 2017; Sarhan & Lilien, 2014; Sarhan, 2017; Sarhan & Jemmali, 2023; Sarhan, Jemmali & Ben Hmida, 2021; Sarhan, 2023). Pan-European privacy-preserving proximity tracing (PEPP-PT) (Rimpiläinen, Thomson & Morrison, 2020), Blue trace, and robust and privacy-preserving proximity tracing protocol (ROBERT) (Aisec, 2020) are the most common crisis-based app protocols that rely on the centralized model.

On the other hand, in the decentralized deployment model (Sarhan & Carr, 2017), the data is owned and controlled by data owners *via* their smart mobile devices. Decentralized models eliminate the drawbacks of centralized models, such as centralized data processing, storage, and computations. No data is supposed to transfer to a centralized server or database for further actions. However, most of the current decentralized protocol relies on a centralized server at one point or the other. The most common protocols that rely on the decentralized model are the Apple-Google protocol (Michael & Abbas, 2020), distributed privacy-preserving proximity tracing (DP-3T) (Troncoso et al., 2005), and the privacy-sensitive protocol and mechanism for mobile contact tracing (PACT) (Chan et al., 2020). For example, in Google and Apple, data is not stored in a centralized database but on people’s phones. Finally, Contra Corona (Bay et al., 2020), Epione (Trieu et al., 2020), and DESIRE (Bielova et al., 2020) are examples of hybrid-based protocols that combine both centralized and decentralized solutions.

#### Cross-border privacy-preserving apps

Since mobile phones have become ubiquitous, they have become an essential tool for data crisis management, so effective collaboration can be performed to respond to a crisis. Therefore, collecting appropriate mobile phone data, including the data gathered by service providers, mobile apps, and embedded sensors, is a required input for practical crisis management tools (Wang et al., 2020). Such behavior, nevertheless, leads to several privacy and security violations.

Interoperability is the primary concern for crisis management, since it has become a critical success factor—Avanzi et al. (2017) proposed a multi-criteria decision analysis (MCDA) method for the public sector to meet interoperability requirements. What we mean by app interoperability is the ability of apps to work together, or to allow integrated operations among different entities to pursue common beneficial goals. An effective crisis management response depends on the level, speed, and precision of information exchanged and the integration of additional services. Enterprise interoperability assessment (EIA) measures the degree of interoperation between entities (Avanzi et al., 2017).

Many crisis management interoperability apps have been deployed to cope with a crisis. For example, KATWARN sends its users warning messages in case of an impending crisis depending on their GPS coordinates (Ion et al., 2020). At the same time, NINA uses GPS or wireless network (Wi-Fi) coordinates to signal its users with warning or recommendations messages (Egele et al., 2013), and other apps like Disaster Alert, Safeture, Facebook Safety Check, Cell Broadcast, SoftAngel, and safeREACH (Grinko, Kaufhold & Reuter, 2019). Despite the criticism received by many crisis based-COVID-19 apps due to the lack of security, privacy, and interoperability, Tauhidi et al. (2022) proposed a privacy-preserved interoperable blockchain-based database for contact tracing and GIS data analysis.

#### Privacy-preserving using privacy set intersection (PSI)

Private sets, or multisets computation, has become popular, and has been in existence for decades since research has worked on improving its computations and communications (Shamir, 1984). It is a cryptography secure, or privacy-preserving computation technique of the intersection, union, and element reduction operations (Kissner & Song, 2005). It was first deployed by Google to securely compute the online advertisement conversion rate (Shamir, 1984; Ion et al., 2020) and later was applied in many applications and scenarios, such as genome tests, Online matching, mobile malware detection service, *etc*.

PSI protects private sets shared by two or more parties by performing a privacy-preserving computation. For example, PSI allows two or more app users to compare their data sets and find intersections without revealing their data. PSI is implemented using many protocols such as public-key, circuit, oblivious transfers (OT), and other variations mentioned in Baldi et al. (2011). Berke et al. (2020) used PSI for contact tracing so users will be informed if they come across a COVID-19-diagnosed candidate. However, their scheme can be practical only if it has been widely adopted (Tamrakar et al., 2017). Trieu et al. (2020) proposed Epione, a PSI-cardinality-based contact tracing app designed to be practical in an intersection between a large server database and a small client one.

### Privacy-preserving using mobile agent

Agent technology has been used extensively in crisis management (Tmnu et al., 2020; Zhou et al., 2021; Kadinski, Berglund & Ostfeld, 2022; Castro et al., 2021). In addition, agent and multiagent systems (MAS) have been used extensively by integrating several emerging technologies to model and provide solutions for complex problems. For example, during the crisis of COVID-19, mobile agent systems have been applied to deal with several issues related to the crisis, for water distribution system contamination response (Kadinski, Berglund & Ostfeld, 2022), to analyze the spread processes of the COVID-19 epidemics in open districts (Castro et al., 2021), and to provide visions for public health policies and interference (Hotton et al., 2022).

Furthermore, Tmnu et al. (2020) proposed a scheme that uses an IoT-based robotic agent for disabled and infected people. The agent uses sensors to identify the patient’s gestures. Finally, Zhou et al. (2021) integrated an agent-based solution with a susceptible-exposed-infected-recovered (SEIR) model to assess the transmission of the COVID-19 viruses inside the city and suggest a vaccine distribution strategy.

### Privacy-preserving based digital health passport

A digital vaccine passport, digital health passport, or immune passport has been widely adopted post-COVID pandemic to respond to the need for resuming international travel. It is a type of official digital document that stores personal information related to individual personal information, including travel history, health information, vaccination status, and diagnostic tests (Angelopoulos, Damianou & Katos, 2020). Thus, the carried data must be anti-fraud, interoperable, privacy-preserved, and manageable (Karopoulos et al., 2021). Many digital health passport solutions (Angelopoulos, Damianou & Katos, 2020; Brown et al., 2020; Gao et al., 2022; Rashid et al., 2022) have been proposed during the COVID-19 pandemic to deal with travel policies, and other restrictive policies introduced during the pandemic.

Most of the proposed privacy-preserving underlying technologies solutions rely on traditional practical public key cryptography and blockchain technologies. However, they encounter many issues due to their implementation or deployment models. Hicks et al. (2020) proposed a decentralized-based public key cryptography scheme called “SecureABC” for immunity certificates. Bansal, Garg & Padappayil (2020) presented a blockchain-based immunity certificate that protects end-users privacy and store testing-related facilities and hospitals.

Electoral commission introduced the idea of implementing a standard for interoperability in the EUROPEAN PARLIAMENT. As a result, a public key infrastructure (PKI)-based digital COVID certificate (EUDCC) presented by the European commission includes the following features: (i) digital and/or article format, (ii) uses QR code, (iii) free of charge, (iv) bilingual, (v) safe and secure (vi) interoperable in all EU countries. Furthermore, such interoperable digital passport permits free movement within European countries (World Economic Forum, 2020).

Furthermore, the idea of protecting against fraud through tests and certificate validation processes is proposed by the CommonPass platform. The platform also validates if the digital certificates are acceptable for international cross-border entry requirements (AOKPass, 2023). AOKpass is a blockchain-based digital passport scheme introduced to enable cross-border interoperability in which users can officially provide digital and authenticated credentials through a QR code to a government authority (AOKPass, 2023).

In addition, Gao et al. (2022) proposed an immunity passport scheme that relies on dual-blockchain architecture using searchable encryption, anonymous authentication, and a decentralized storage network using the inter planetary file system (IPFS). While the scheme provides data privacy for the users and ensures unrestricted movement as claimed by the users, unlike our scheme, their proposed solution is not smart. It only shares immunity passport data but not other sensitive data. Furthermore, the solution relies on IPFS to store encrypted passport data, which is impractical for storing private data (Gao et al., 2022). Koyama et al. (2023) proposed a decentralized vaccine distribution scheme to track the vaccine route. The scheme relies on Blockchain as an underlying technology and consists of three components, including a “vaccine passport.” It is not fully practical compared to our scheme, and it has many limitations, as pointed out by the authors (Koyama et al., 2023).

## Crisis-based privacy-preserving apps systematic review

This section provides a brief evaluation study that highlights the pros and cons of the current existing platforms, and compares them with the proposed scheme. Due to climate change, widespread diseases, and unpredictable disasters, which cause life losses, economic damages, and privacy and security violations, many tools have been developed to cope with such issues.

However, despite the numerous advantages gained by such tools, their contributions only minimize the impact of the incidents. In other words, no single solution is considered fully practical to tackle most problems. The common drawbacks of the proposed solutions are as follows: (i) data security and privacy leakages, (ii) failure to comply with international privacy and data protection standards, (iii) surveillance, (iv) sharing data with trusted and untrusted parties, (v) poor functionality, (vi) limitation in computational power, (vii) untrusted deployment model.

Table 1 shows a concise evaluation that compares different crisis-based management apps to address most problems affecting them. Table 2 shows the acronyms used in Table 1 with the corresponding terms. Although this research aims to cover issues pertinent to digital health passports, the researcher reviewed thirty-six applications deployed in various domains, such as immunity passports, contact tracing, and monitoring, as shown in Tables 1 and 2. The evaluation criteria considered many factors, such as data privacy and security, deployment models, underlying technology, privacy protection complaints, domain, interoperability, and level of sensitivity of data.

**Table 1:** Crisis apps major comparison, features, and privacy-preserving underlying technologies.

Platform	Domain		Deployment model		Protocol		Underlying technology			Data sensitivity level	Mandatory data minimization	Privacy violation level	Possible target attack risks severity factor	Interoperability
IO app	Immunity passports										OPT.	S	2	
CovPass			Decentralized							3		L	11	EU
Surokkha app		Cent.									No	S	2, 14	NO
MyCOVID Pass														AU
SMDPA		DHDP			Agent-based PSI (PKI)					N/A	Yes	Low	N/A	Yes
Blockchain.plat.							Blockchain							EU
Coronapas app					PKI						N/A	Severe	14	No
Tawakkalna		Response based system	Centralized				Bluet.	GPS		1, 5, 6	NO		1, 14, 15	
Chinese Alipay	Contact tracing (CT)						AI			1, 3			2, 3, 14	
ENACT							Wi-Fi-						2, 3	
TraceTogether					BlueTrace		Bluetooth			1, 2		2, 3	12, 14	
COVIDSafe										1		M	5, 14	
ABTraceTogat.										4		Severe	14	
Aarogya Setu								GPS		4, 5			1, 14, 15	
REACT							BLE			5			2, 3, 14	
Conrona-Korea		Self-D								1, 2, 5			2, 3	
Iranian AC19			Decentralized							1, 5			2, 3	
Apple & Google		NT			Apple & Google		Bluetooth			N/A			4, 13, 14	Yes
NHS-COVID19										3		Low	2, 3, 4, 12, 13	No
SwissCOVID						DP-3T					1		6	
CoronaWarn					PEPP-PT						Yes		14	
USA Safepaths									GPS		NO		2, 3, 12, 15	
COVID-19 KP												Severe	12, 15	
WeTrace										3, 6			2, 3, 14, 15, 16	
LeaveHomeSafe							AI						11	
Blockchain-based-magnet.							Magnet.					L	19	
WifiTrace							Wi-Fi					S	15, 1, 7, 8, 9, 10, 2, 3	
MagnetomTrace							Magnet.					M	15, 16	
PTBM							5G-Based-Blockchain							
RFID-based CT							RFID					S	17	
IoT-based-CT	MN *FU*					IoT						S	1, 15	
SDN-Plat.	Telemedicine services		Cent.				SDN					Medium		
IoT.SDN-Plat.	Monitoring							IoT					18	
RSSI-based-SD	Social distance (SD)		N/A				Bluet.		RSSI	8			13, 14	
RSSI-based-SD								ML		8			13, 14	
TraceScan	RAS		HYB.						4, 7, 8	NO	L	2, 3		

**Table 2:** List of acronyms for feature terms in Table 1.

Acronym	Term	Acronym	Term
AI	Artificial intelligence	GPS	Global positioning system
RSSI	Received signal strength indicator	Wi-Fi	Wireless fidelity
SDN	Software-defined networking	RFID	Radio frequency identification
IoT	Internet of things	ML	Machine learning
5G	5th generation mobile network	Magnet	Magnetometer
Bluet.	Bluetooth	OPT.	Optional
NT	Notification systems	MN	Monitoring
FU	Follow up	Self-D	Self-diagnosis
RAS	Risk alerting system	DHDP	Digital health and data passport
BLE	Bluetooth low energy	PKI	Public key infrastructure
PEPP-PT	Pan-European privacy-preserving proximity tracing	DP-3T	Distributed privacy-preserving proximity tracing
HYB	Hybrid	Cent	Centralized
AU	African union	EU	European union
S	Severe	M	Medium
L	Low		

The presented comparative study is based on the platform’s domain, deployment models, underlying technology, privacy and security violations, threats, and interoperability. The evaluation of the deployment model shows that the decentralized model is far better than the centralized one. Centralized-based platforms rely on the central server design, which has received much criticism, because of their serious privacy violations. TraceTogether in Singapore (Singapore Government Blog, 2020) and Canada, AarogyaSetu in India, ENACT and COVIDSafe are examples of centralized-based design solutions (Lodders & Paterson, 2020; Norton Rose Fulbright, 2020; MyGov, 2020; Prasad & Kotz, 2017). They have been built to assist in coping with crises, seeking treatment, and helping people accomplish everyday activities promptly. Thus, such solutions pose many drawbacks, including data leakages, surveillance, and side-channel attacks. On the other hand, decentralized-based protocols have been adopted to tackle the issues presented in TTB-based ones.

Decentralized protocols have gained privacy and security advantages by allowing users to store and manage their data on their mobile devices without interacting with the trusted third-party server (TTP). It relies on distributed storage or servers. It protects identities against untrusted parties and protects data against exposure. For example, SwissCOVID, Safepaths in the USA, WeTrace in the Philippines, CovPass, and CoronaWarn (von Wyl et al., 2020; Raskar et al., 2020; Gassmann, 2020; Hernández et al., 2021; Reelfs, Hohlfeld & Poese, 2020) are platforms built based on decentralized design. Apple–Google, BlueTrace (Bay et al., 2020), DP-3T, and PEP-PT are examples of the popular decentralized protocols that should overcome issues presented by the centralized ones. Nevertheless, several platforms built based on such protocols have been vulnerable to security and privacy flaws, health data leakage, GDPR compliance issues, replay attacks, and trust (Wymant et al., 2021; Messai & Seba, 2020).

Furthermore, some solutions considered combining both models to develop a hybrid approach to building apps relying on centralized and decentralized protocols; for example, CT-RSA (Srithas & Navaratnam, 2020). Yet, as shown in Table 3, such apps are vulnerable to surveillance, man-in-the-middle (MITM) attacks, and key recovery issues. Table 4 shows a score for the data sensitivity level, which explains the severity of data leakages, with one being the highest and eight the lowest level of sensitivity.

**Table 3:** Privacy-preserving risks/threats impact level.

Name of issues	Risk impact factor	Name of issues	Risk impact factor	Name of issues	Risk impact factor
Security & Privacy flaws	1	Key recovery	8	Single point of failure	14
Sensitive data leakage	2	Denial of service	9	Poor functionality	15
Health data leakage	3	Traffic description	10	Drain battery	16
Surveillance	4	QR code leak	11	Storage limitation	17
Replay attack	5	Data sharing with TTP	12	Require technical skills	18
Linkage attacks	6	Fail to comply with privacy Act	13	High installation and operation cost	19
Man in the middle	7	Profiling	14		

**Table 4:** Data sensitivity levels for popular crisis apps.

Category of leakage data	Data sensitivity level	Category of leakage data	Data sensitivity level
Personal & Identities data	1	Location data	5
Health data	2	Device ID	6
QR code	3	Time	7
Bluetooth ID	4	Distance information	8

Evaluating the apps listed in Table 1 based on ethical and data protection principles showed that none fully complied with international data protection acts. For example, platforms such as Hernández et al. (2021), Trusted Travel (2021) only comply with data protection standards inside the European Union countries. Furthermore data, Besides privacy and security concerns, other challenges have been presented when evaluating the selected platforms based on functionality, performance, computing resource usage, complexity, and usability. For example, AarogyaSetu, WeTrace, Safepaths, and COVID-19 KP showed poor functionality. Moreover, WeTrace, and Magnetometers Trace (Kuk, Jeon & Kim, 2017) experienced drain battery issues. In addition, RFID-based contact tracing (Mehta et al., 2020) encountered storage limitations. Other platforms (Jung & Agulto, 2021; Jeong, Kuk & Kim, 2019) struggled with technical and training skills requirements and operation complexity.

This evaluation intends to select applications relying on different underlying technologies such as GPS, Bluetooth, BLE, Wi-Fi (Trivedi et al., 2021), Machine learning, magnetometer, RFID, RSSI, cellular network (5G), IoT (Roy et al., 2020), Blockchain (Rashid et al., 2022), SDN, and Machine learning. Therefore, the researcher noticed that most selected platforms relying on GPS as an underlying technology experienced sensitive data and health leakages, such as REACT (Xiong et al., 2020), Iranian AC19 (Messai & Seba, 2020), and Apple-Google (Wymant et al., 2021). Moreover, platforms relying on cellular networks, Wi-Fi, GPS, or Bluetooth recorded severe data privacy violations. Only a few offered an optional data deletion feature, for example, CovPass, Surokkha (Mary et al., 2023), and IO platforms (IO, 2022).

Another evaluation intends to evaluate the platforms regarding interoperability and privacy protection act compliance. For example, we observed that only an international application like the one jointly built by private companies Apple-Google (Apple Google, 2020; Michael & Abbas, 2020) could practically function worldwide to overcome cross-border app interoperability. Such a platform, nonetheless, has raised serious concern among French parliamentarians (Storeng & de Bengy, 2021), pointing out that it could be used to share and sell health data, including digital sovereignty. Other applications like the CovPass and the blockchain-based ones are only interoperable in European countries (Hernández et al., 2021; CovPass, 2021). Furthermore, MyCOVID Pass (My Covid Pass, 2021) operates interoperability only inside African countries.

### Other solutions *vs* CONTRIBUTIONS OF THIS ARTICLE

The proposed secure mobile digital passport agent (SMDPA) includes the following features: It (i) securely shares personal and health information with international authorities; (ii) uses a mobile agent to disseminate data associated with their security and privacy policies; (iii) supports international privacy standards and regulations *via* the use of intelligent data minimization feature; (iv) uses privacy set intersection technique to provide confidentiality and integrity of the carried data and relies on a mobile agent fault tolerance feature to support data availability; (v) uses data evaporation feature to expire health vaccination information when applicable; (vi) supports interoperability to relax international travel; (vii) protects against discrimination by providing anonymous, secure interaction between users and authorities so limited information can be shown (viii) provides recommendation for safe travel zone based on a traveler stored health information and general health conditions.

Unlike other digital passport protocols, our protocol combines the following core features: (i) interoperability, (ii) fit privacy standards and regulations, (iii) fault tolerance, and (iv) data minimization.

## Problem statement

**Figure 1:**
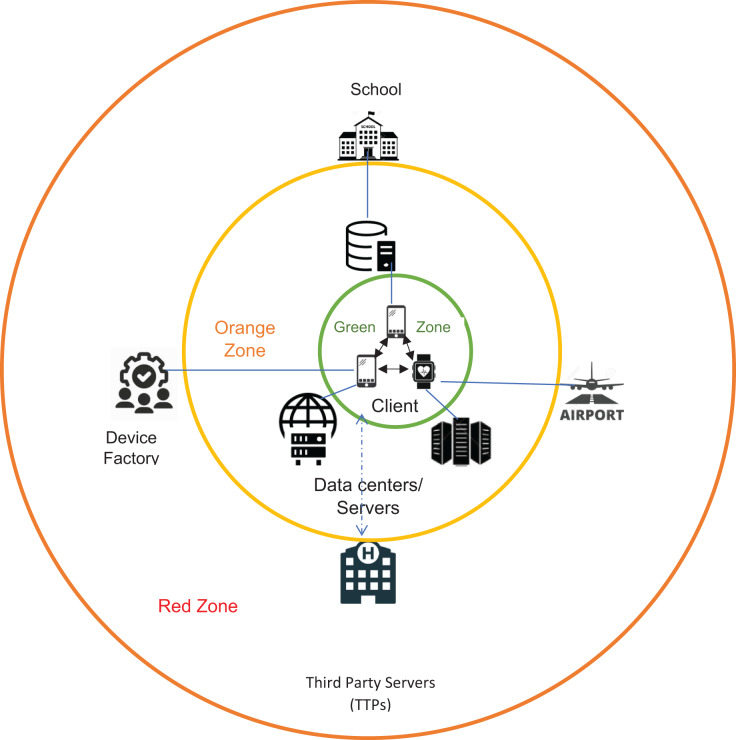
General architecture of current crisis apps platforms interaction.

Current digital health passports, immune passports, or vaccine passport apps include limited health information that neither can be shared anonymously (due to massive surveillance) nor grants an individual an ideal free movement or be processed autonomously. This problem can be modeled as a privacy set intersection where two mobile agents can represent two parties to securely compute the intersection of digital health passport data and institutional distributed servers or databases datasets. As discussed previously, secure mobile digital passport agent (SMDPA) is a digital health passport mobile agent that directs its owner to mobilize according to the intersection results between SMDPA and the institutional distributed databases or servers agent. Figure 1 shows a client-server architecture of the most common crisis-based applications which rely on trusted third-party servers (TTPs). It classifies the risk of leaking information into three levels or zones: green, orange, and red. The green zone shows a peer to peer communication between devices in which each owns its data. However, if such devices share data with internal TTP, such as data centers, the chances of leaking the information increase. The red zone would show a high probability of leaking information if such devices shared information with external TTPS without permission.

This research defines a digital health passport as one holding an individual’s personal information and health information. The information includes medical health records, including conditions, infections, symptoms, medical drug lists, vaccination status, and risk factors. Unlike many proposed digital health passport solutions, SMDPA, as an intelligent agent, interacts autonomously with other parties (*e.g*., other agents) on behalf of its owner in a decentralized manner. This should overcome issues inherited from a client-server model concerning internet traffic and bandwidth overhead. Moreover, its privacy policy involves a data minimization function that deals with cross-border data privacy regulation and standards compliance.

Let *M* be a party owning a set of private information concerning an individual’s personal and health information. Let *A* be an authority, institutional, service provider, or governmental agency holding encrypted information stored in distributed databases. *M* and *A* want to apply an exact join to their data without revealing unnecessary information. This means that the only information learned by *M* about *A* and information retained by *A* about *M* is M $\cap$ A. Let’s assume *M* is a source containing a set of elements (m_1_, m_2_, m_3_, …., m_n_), and *A* has (a_1_, a_2_, a_3,_ …., a_n_). Private set intersection (PSI) can be used if both parties want to apply to join on their private sets without revealing any data except the elements in the intersection data.

This research designs a protocol in which the datasets *M* and *A* obtain the intersection under privacy constraints, which states the protocol must not reveal elements in the intersection. Furthermore, the proposed protocol avoids relying on a trusted third-party server (TTP) to compute the intersected elements between *M* and *A*. Instead, it is a decentralized protocol that relies on a mobile agent as an autonomous entity to act on behalf of the travel passenger when interacting with other parties.

The researcher assumes that international cloud repositories, or distributed databases, are deployed, decentralized, and managed based on multiagent systems (MAS), and hold information concerning crises, including health conditions and requirements. For example, an institution party (such as a hospital or a school) can update this repository’s information (*e.g*., local lockdown, restricted and green zones). Such shared information can benefit passengers using SMDPA.

The current proposed protocol uses PSI to allow SMDPA users to compare their digital health passport set of elements (*M*) with the data stored in an internationally distributed cloud database server (*A*) without revealing any information concerning their privacy. Hence, PSI allows SMDPA users to check whether their digital health passport data and privacy policy (*M*) intersect with data and privacy policy stored in “*A*,” a distributed database, without revealing *M* datasets.

Although unbalanced PSI (Davi Resende & De Freitas Aranha, 2021) seems the best to suit the proposed approach in this article; yet, this article does not focus on the implementation, or modification aspect concerning PSI, which is left for future investigation. Furthermore, the intersected data sets are not balanced because the data elements in the digital passport represented by SMDPA contain a limited dataset compared to the one stored in an institution’s server.

**Figure 2:**
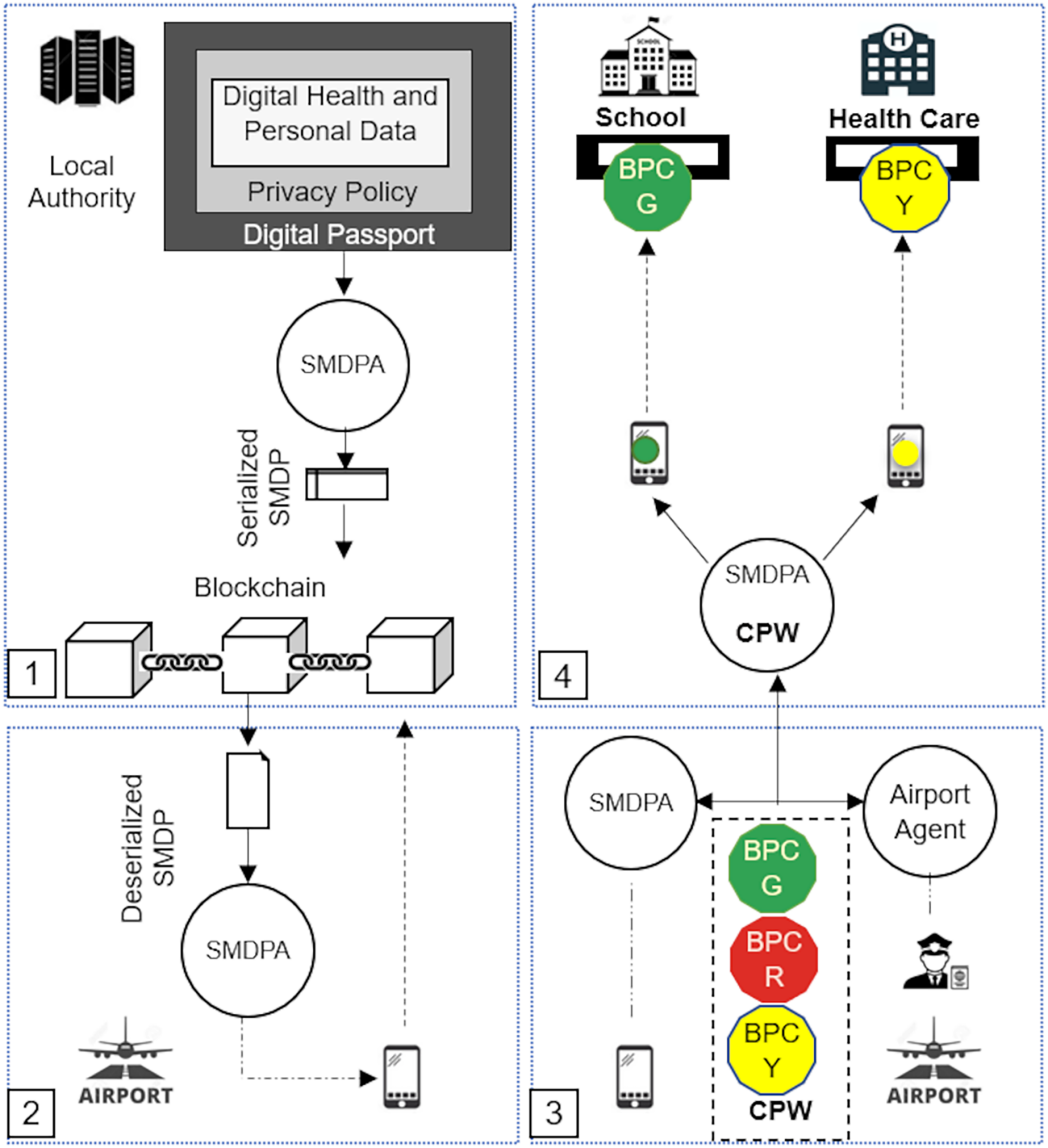
Secure mobile digital passport agent (SMDPA) high-level architecture.

Figure 2 shows a high-level architecture of the secure mobile digital passport agent (SMDPA) solution. The top left side of the figure, labeled 1, shows the process of creating SMDPA. First, the health, personal data, and privacy policy encapsulated to form a digital passport which is then serialized in a local authority ledger. The bottom left side of the figure labeled 2 shows the process of deserializing the SMDPA which is invoked to be carried by a mobile agent. This step occurs when the passenger is about to leave the local airport. The bottom right side of the figure, labeled as 3, shows the process of datasets intersection at the destination or the international airport between SMDPA and the airport agent. The intersection results should generate *n* bit passing coin (BPC)s stored in a coin passing wallet (CPW) and carried by SMDPA. Finally, the top right side of the figure shows SMDPA trying to access a restricted area in which one BPC of a particular color is demanded to grant access. The figure shows peer-to-peer communication in which the information is shared in a MAS environment where the chances of leakage are low compared with the architecture presented in Fig. 1.

## Architecture and design of the proposed scheme

### Protocol design

The protocol security design in this research relies on public-key cryptography based unbalanced-private set intersection (PSI). The protocol deals with unbalanced datasets since the data carried by the secure mobile digital health passport agent (SMDPA) is less than those stored in an institutional distributed repository. Hence, despite many existing PSI protocols, a one-way PSI protocol seems the best to fulfill the requirement in this research; therefore, only SMDPA should know the intersection result. Bloom filter (Bloom, 1970 ), Cuckoo filter (CF) compressions, Cuckoo hashing (Fan et al., 2014), original quotient filter (QF), or rank and select based quotient filter (RSQF) (Pandey et al., 2017), can be integrated with the one-way PSI to decrease the amount of transmitted data or stored data by SMDPA. Measuring the best filter that suits our protocol’s design is outside this article’s scope and plans for future work. The setting of our protocol is as follows:

1. Use unbalanced PSI since we assume that one party has a set with 10 or few hundred of data (SMDPA) and the other party might have a set with a few million to billion data records.
2. Assume a one-way PSI protocol to interact with the server agent to minimize the amount of overhead inherited by the two parties (mutual).

Assume a PSI-based enrichment scenario since both parties, the SMDPA and the server agent, want to (i) apply joint between their datasets without revealing any unnecessary information and (ii) enrich joined records with variables from both SMDPA and the server agents. For example (see Table 5), Given set A = {age: 8–60, 4–80, 17–50, 17–45; DH: COVID-19, Ebola, Type2 Diabetes, Hepatitis C; HR: one-dose, two-doses, quarantine, health insurance; GZA: USA, Germany, KSA}, and set M = {PN: p12, age: 32, DH: COVID19, HC: one-dose, Date: 1/1/2023, TH: China, USA, KSA, UAE}. Thus, M ∩ A = {P12, 32, USA, Germany, KSA, Mall, Restaurants, Hospitals, one-dose, 1/1/2023, China, USA, KSA, UAE}. An example of elements that should remain outside the intersections {Nationality, Religion, and Travel history}; such information can be subject to discrimination, refusal of employment, social media, racial, religious profiling, advertisements, or scams.

**Table 5:** Privacy set intersection based on enrichment case.

Passport number (PN)	Age	Disease history (DH)	Health requirements (HR)	Green zone airports (GZA)	Red zone airports (RZA)	Green zone places (GZP)	Yellow zone places (YZP)		Red zone places (RZP)
P_n_	8–60	COVID-19	One dose	USA Germany KSA …..	China Switzerland Ukraine ……….	Mall Restaurants Hospitals ……………	Schools		Kindergarten
	4–80	COVID-19	Two doses	USA Germany KSA Switzerland	China				Children’s Park Zoo Kindergarten ………..
	17–50	COVID-19	Quarantine	China	China				Children’s Park Zoo Kindergarten ………..
	17–45	Ebola	Quarantine	USA	China KSA				Children’s Park Zoo Kindergarten ………..
	40–100	Type 2 diabetes	Health insurance	USA Switzerland	None				None
	17–45	Hepatitis C	Two doses	All countries	None				None
	Passport number (PN)	Age	Nationality	Religion	Disease history (DH)	Health conditions (HC)	Date	Travel history (TH)	Institution *A*
SMDPA *M*	P_12_	32	USA		COVID-19	One dose	1/1/2023	China USA KSA	
		P_12_ 32	COVID-19	USA Germany KSA Mall Restaurants Hospitals……	China Switzerland Ukraine Kindergarten………………				

The goal of this protocol is to prove eligibility while hiding an individual no essential identity. While several existing PSI protocols and variations encounter many computational and communication overhead issues, SMDPA should overcome communication and computation overhead as a mobile agent. An agent can allow code and data to carry their security or protection mechanisms wherever they travel. This improves traditional security solutions, where a stationary platform manages security and protection. Let us consider the following examples. An immigration and immunization service department or health care agency:

1. Want to ensure that passengers have no severe health cases so that they can be allowed entry but denied or directed to an international event. Neither the passenger nor the agency wants to disclose their data, but both want to know the intersection.
2. Compare their databases of common health diseases with tourists while respecting international and local privacy laws that prevent them from exchanging or revealing information. Thus, they can share minimum allowed information related to subjects of interest matter.
3. Identify visitors who visited countries with high infection rates without identifying the countries or placing travel restrictions.
4. Check its database of hazardous diseases against foreign air carrier-passenger digital health passports without both parties revealing their set of data. Such passengers might be denied flying into a particular restricted zone.

To design the PSI protocol, the following points are to be taken into consideration:

1. The size of the dataset in both parties. For example, the size of *M* and *A*. SMDPA datasets M is expected to be small compared to those owned by an institution or interacted agency.
2. The level of privacy and security needed to tackle any adversarial attacks.
3. The resource-constrained or computational power for smart mobile devices since multiple cycles of interactions are not recommended. SMDPA is not required to download large datasets nor perform an intensive computation that might drain the battery.

### Example 1

In this example, let’s assume there are two datasets. Set A contains private data that are encoded as integers and have {0, 5,10, 15, 20, 25, 30, 35, 40, 45, 50}, and Set B includes information related to site restriction and health requirements that are also encoded as integers as follows {0, 4, 8, 12,16, 20, 24, 28, 32, 36, 40}. So set A $\cap$ Set B = {0, 20, 40} and hence the intersection size (*IS)* is 3.

We assume that *IS* a factor that determines the place of visit for an individual in a crisis-based situation. Based on *IS* and the intersection matching result (*IMR*) values, three levels of bit passing coin (BPC) are generated. BPC is a single access permit value that permits an individual to access an institutional area (say, an airport, hospital, school, *etc*.).

Each level of BPS is represented by a color described as follows: (i) green: an individual is fully permitted to enter any place in the green zone based on his health status determined by the set intersections. BPC_G_ denotes BPC passing for green zone areas. (ii) Yellow means an individual can access the yellow zone area. BPC_Y_ symbolizes BPC passing in yellow zone areas. (iii) Red indicates an individual is permitted to access the red zone area. BPC_R_ implies a passing permit in red zone areas. *IMR* contains interesting elements describing specific medical and personal data. Note that the number of generated BPCs varies from person to person, considering individual health and personal information such as medical history, age, vaccination, *etc*. Therefore, it depends on a particular health condition. There is a threshold *TH* value that manages the generated BPC. *TH* categorizes BPC into three levels described above, which are represented as Level 1 (L1), Level 2 (L2), and Level 3 (L3), as shown in Algorithm 1, Table 6.

**Table 6:** SMDPA algorithm description.

Algorithm 1. *SMDPA* Algorithm
A = SMDPA dataset elements
B = AgentServer dataset elements
C = A $\cap$ B
IS $\leftarrow$ Size of C
TH $\leftarrow$ Threshold L1, L2, L3
CPW $\leftarrow$ store Coin Passing Wallet (BPC_G_, BPC_Y_, BPC_R_)
**if** (IS $\geq$ L1) **then**
Generate n BPC_G_
_ _Add BPC_G_ to CPW
**else**
** if** (IS $\gt$= L2 && IS $\lt$ L1) **then**
Generate n BPC_Y_
Add BPC_Y_ to CPW
** else**
** if** (IS $\gt$= L3 && IS $\lt$ L2) **then**
Generate n BPC_R_
Add BPC_R_ to CPW
** end if**
** end if**
**end if**
Calculate CPW
Return CPW

### Example 2

Let us assume a scenario where *IS* & *IMR* indicates an individual can visit the green zone area, assuming *IS* & IMR $\leq$ L1. In this case, an individual is granted nBPC_G_ to be deposited in his coin passing wallet (CPW) as n(BPC_G_). This means he can access only n green zone areas daily. Note that the number of generated BPCs depends on other factors, such as individual health, data records, and vaccinations. It is specifically determined during the first privacy set intersection, which is assumed to be at the airport’s first entry point. Let A be an encoded dataset of ten elements {1, 2, 3, 4, 5, 6, 7, 8, 9, 10}, B encoded dataset of nine elements {0, 1, 3, 4, 5, 6, 7, 8, 9, 10}. A $\cap$ B = {1, 3, 4, 5, 6, 7, 8, 9, 10}. Assume the threshold TH sets its first level L1 to be nine or more for a green zone. In this case, n BPC_G_ is generated and deposited into CPW since *IS* $\leq$ *L1, (*9 $\leq$ 9 *)* and *IS* {elements} € IMR. TH can also be arranged to generate the number of allowed BPCs for L2 and L3, described as the yellow and red zones.

### Algorithms description

Tables 6 and 7 show the algorithms presented in this scheme. Table 6 algorithm is described as follows: (i) The result of the sets intersection size of the secure mobile digital passport agent (SMDPA) and the airport agent stored in variable *IS*. (ii) There are three levels of threshold presented as L1, L2, and L3 such that L1 is the largest, L2 the second largest, and L3 the lowest. (iii) Using the random number generation function to generate n BPC, then getting stored in CPW according to the three branching logic to determine the order. The logic compares the largest, median, and smallest threshold with the set intersection size. It generates n BPC because the larger the intersection, the more BPCs will be generated to be stored in CPW. CPW is modeled as an ArrayList object. Table 7 shows algorithm 2. It presents an enhancement for algorithm 2. It takes the average of L1 and L2 and compares the result with *IS*. Else takes the average of L2 and L3 and compares the result with *IS*.

**Table 7:** Enhanced SMDPA algorithm description.

Algorithm 2. Enhanced *SMDPA* Algorithm
A = SMDPA dataset elements
B = AgentServer dataset elements
C = A $\cap$ B
IS $\leftarrow$ Size of C
TH $\leftarrow$ Threshold L1, L2
CPW $\leftarrow$ store Coin Passing Wallet (BPC_G_, BPC_Y_, BPC_R_)
**if** (IS $\gt$ (L1 + L2)/2) **then**
Generate n BPC_G_
_ _Add BPC_G_ to CPW
**else**
** if** (IS $\lt$ (L2 + L32)/2) **then**
Generate n BPC_Y_
Add BPC_Y_ to CPW
** else**
Generate n BPC_R_
Add BPC_R_ to CPW
** end if**
** end if**
**end if**
Calculate CPW
Return CPW

To accelerate Algorithm 1, we used the dichotomy strategy to find the middle point between L1 and L2 and then compare the result with the intersection size *IS*. We use dichotomy to determine the optimal threshold value that speedup up the comparison process between intersection size and the set thresholds. The dichotomy is used for sorting, but we use it in our algorithm due to its success in solving other problems. We set L1 = 17 and L2 = 12. The proposed protocol assumes that the set stored at the airport authority is static. Hence to prove the proposed scheme’s concept or practicality, the size of the ArrayList that represents the airport’s authority or data repository was 23 values encoded as integers. The size of datasets sent by SMDPA was between 1 to 5. We did not implement the encryption and decryption process. However, we simulated the set intersection using two agents one is SMDPA, and the other is the Agent that represents the airport authority.

### System model

**Figure 3:**
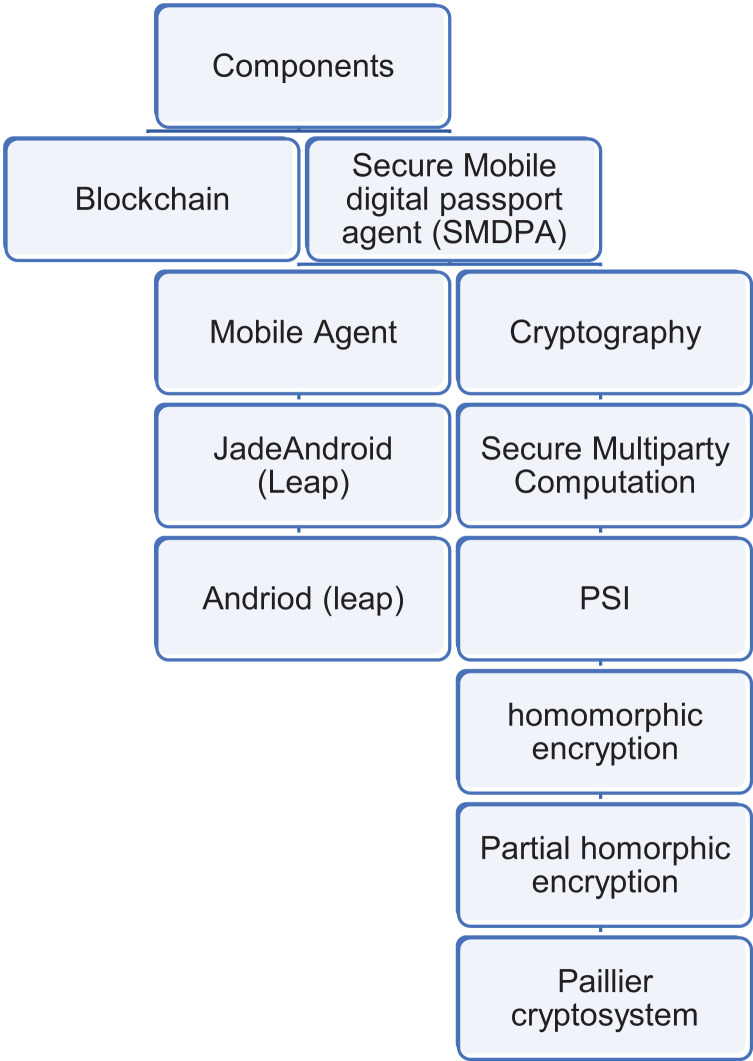
The component of SMDP solution.

This sub-section presents the components of the proposed SMDPA solution (see Fig. 3).

*(1) Secure mobile digital passport agent (SMDPA)*: is a software construct based on a mobile agent that encapsulates data and its privacy and security operations policy. The proposed scheme modified the solution proposed in Sarhan & Carr (2017) as follows: (i) private set intersection (PSI) is employed as a data protection scheme that also manages the privacy access policy and data evaporation. We assume the privacy policy contains two attributes labeled as time and location to control the trigger of the data minimization procedure in a specific time and geographical location. This should deal with issues related to privacy compliance. To balance the CIA-Triad, a self-destruction feature (Sarhan & Carr, 2017) was excluded as we feel that such a powerful feature is against the data security policy in maintaining data availability. The proposed solution inherits the data evaporation feature presented by Othmane & Lilien (2009), which we call data minimization. Data evaporation or data minimization is an essential feature of the proposed scheme. However, it is outside the scope of this article to discuss data evaporation in detail. In the Sarhan & Carr (2017) scheme, attribute-based encryption is used, so the logical network location and actual time are labeled as location and time attributes. Hence, the data evaporation was applicable when a specific time or location met certain conditions. The private set intersection scheme used in this article needs to be reconstructed to guarantee data evaporation while ensuring data privacy. For example, Sparka, Tschorsch & Scheuermann (2018) proposed a P2KMV algorithm that performs set computations to provide set intersection cardinality and data minimization.

#### SMDPA-sub-components & features

*(1a-1) Java agent development framework (JADE)*: Jade is an open-source agent framework with numerous built-in and add-on functions and libraries. It can be utilized to develop distributed applications, support the J2ME platform and wireless environment, and provide decentralization environments in many operating systems. Its rich communication protocols can provide inter-platform and intra-platform messaging (Bellifemine, Caire & Greenwood, 2007).

*(1a-2) Jade with lightweight extensible agent platform (Leap) Add-on*: Jade Leap is a multiagent systems (MAS) environment combined with Jade to support mobile phones.

*(1a-3) Java J2ME:* Java 2 Platform, Micro Edition or (J2ME) is a Java version or edition designed to address limitations on the application running on embedded systems and mobile devices with limited processing power and memory. Many devices support J2ME because it is simple and easy to implement. It is used for portable code for embedded and mobile devices.

*(2a) SMDPA-*Security policy: SMDPA, like active data bundles using secure multi-party computation (ADB-SMC) (Sarhan & Carr, 2017), encapsulates a privacy and security policy with the digital health passport data. The policy protects and controls digital health passport data’s security, privacy, and anonymity. In addition, it controls how data are being intersected and minimized when interacting with other parties. The decentralized cryptographic protocol that protects data is described next:

*(2a-1) Private set intersection (PSI)*: SMDPA protects its data using PSI, a robust, secure multiparty computation or privacy-preserving protocol that makes two parties compute the intersection of their data and output only the intersected data. The purpose of using PSI is to share and process data anonymously between two parties and guarantee flexible control of the movement of individuals during a crisis. For example, travel passengers might be directed partially to visit certain areas and restricted from entering others. PSI can ease travel while providing anonymity for the passengers. This should deal with profiling or any form of discrimination concerning race or other discriminatory cases. For example, Asian Americans have experienced anti-Asian discrimination fueled by the crisis of COVID*-*19 (Gover, Harper & Langton, 2020). Also, the SMDPA policy uses two attributes for privacy minimization service, described next.

*(2a-1-a)* *Homomorphic encryption (HE)*: is a secure public key (PKI) encryption scheme that makes two parties perform computation on encrypted data. This should ensure privacy for the datasets where only one side needs to encrypt and decrypt. He is beneficial, especially in the case of multiple parties do not exist, or there were a limited number of participants to use secret sharing for secure multi-part computation as the case in our protocol.

*(2a-1-b)* *Paillier cryptosystem*: is a partially homomorphic encryption that permits two kinds of computation on ciphertext: (i) addition and (b)multiplication. The proposed protocol is extended based on the Paillier cryptosystem to reveal only the intersection size for SMDPA. In addition, both SMDAP and the server agent perform private cardinality matching. More detail is found in Freedman, Nissim & Pinkas (2004).

*(2a-2) Time attribute:* SMDPA is assumed to use time attribute to deal with specific lockdown scenarios or travel policies. The time attribute can be used as an example to remove any travel data restriction concerning vaccination against certain diseases. For instance, post-COVID-19, some countries imposed travel requirements for air passengers that requested travelers to wait 14 days after a specific dose of vaccine (CDC, 2019).

*(2a-3) Location attribute*: SMDPA is assumed to use location attributes to deal with travel policies imposed by some geographical regions and privacy policies like the general data protection regulation (EU GDPR), which address data transfer outside the EU. For example, the SMDPA data minimization feature can evaporate data concerning individual health status and data privacy under specific time and location requirements.

*(2a-4) Bit passing coin (BPC):* BPC is an idea that is presented from the coin vending game machine. It states that the result of a set intersection between SMDPA and the entry point (airport) distributed repository server agent should generate BPCs in three colors: Green, yellow, and Red. For example, Green BCP should permit a person to move freely and access a protected zone during a crisis. Yellow BCP should allow a person to pass through a particular area. Red BCP should restrict an individual from passing through most of the area and only access the effectively protected zone. Each person crossing a border should receive several BCPs in various colors. The BCPs are determined based on the crisis conditions.

*(2) Blockchain*: Blockchain is a peer-to-peer technology based on a distributed ledger. It can record the participants’ activities in its network. It relies on several cryptographic applications, such as encryption, hash functions, and digital signature. In Blockchain, data is signed digitally as transactions and then broadcasted. All broadcasted transactions are timestamped, grouped, and hashed orderly into blocks forming unique identifiers of blocks. Integrating multiagent systems (MAS) into Blockchain has many benefits, including (i) addressing scalability issues in Blockchain, (ii) managing the large datasets stored in the distributed database servers that SMDPA, for instance, has to interact with; and (iii) improving digital health passports and healthcare management; (iv) fixing any security limitations in MAS; and (v) adding more flexibility to MAS (Calvaresi et al., 2018). Details about integrating the proposed scheme with Blockchain are outside the scope of this research. However, for future work, we plan to study the serialization and deserialization of SMDPA agents in the form of a Blockchain. Serialization means turning SMDPA agents into a data format, which can be saved into storage and deserialized where applicable.

**Figure 4:**
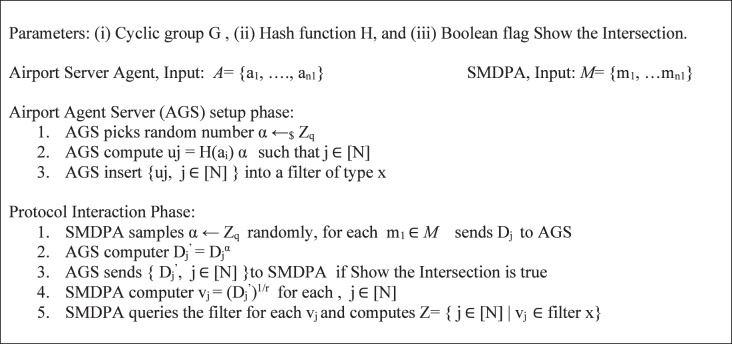
Basic PSI protocol adapted from Angelou et al. (2020).

*(3) Preliminaries*: Fig. 4 shows the basic PSI protocol adapted from Angelou et al. (2020). The protocol combines Diffie-Hellman (DDH), based PSI, and PSI-Cardinality; and uses Bloom filter compression to minimize communication time. The proposed protocol develops set intersection cardinality (PSI-CA) on top of the Diffie-Hellman (DH) because PSI-CA-based DH gives lower communication costs. First, the airport agent server (AGS) and SMDPA agree on a large prime p. Then, the AGS generates private key α, hash its dataset values a1, a2, *etc*., and then calculates (ai)^α^ for each hashed value. Next, the AGS inserts the result “uj” into a bloom filter and then sends it to SMDPA. “uj” corresponds to the item mj (Trieu et al., 2020). On the other hand, SMDPA then generates private key r, hashed its datasets, and then calculates Dj = (mi)^r^ for each hashed value and sends the result to AGS, which uses its private key to compute Dj’, (Angelou et al., 2020; Trieu et al., 2020). Finally, this protocol is extended to reveal only the intersection size based on the Paillier cryptosystem. Additionally, using PSI-CA that decides if the intersection size is larger than a pre-specified threshold L is also detailed in Freedman, Nissim & Pinkas (2004).

## Smdpa simulation experimentation

### Simulation setup

Table 8 lists the simulation environment specifications. The secure mobile digital passport agent (SMDPA) system is simulated using a personal desktop with a single processor with 8 GB of RAM. The desktop includes the Java agent development framework (JADE) platform and several add-on libraries described in the previous section. Since JADE cannot function properly on small devices, the LEAP add-on is integrated with JADE. Hence, the Jade runtime environment was modified to form JADE-LEAP that can be deployed thereafter on a wide range of small devices. J2ME Configuration uses either connected limited configuration (*CLDC*) or connected device configuration (*CDC*). Cell phones or PDA device versions can use either technology depending on memory availability. For example, devices with low memory use *CLDC*, and devices with better memory use *CDC*. The researcher used the *CLDC* of Java Micro Edition (J2ME CDC) to form the JADE-Leap. The configuration of JADE-Leap is based on MIDP, which runs on devices that support Java-enabled cell phones. The simulation management of the SMDPA and the distributed server agent is carried out through Agent.GUI. Agent.GUI also records the interaction performance measurements between SMDPA and the distributed server agent (Derksen, Branki & Unland, 2011).

**Table 8:** The configuration of the computing environment for SMDPA.

Hardware specification		
CPU	Intel (R)i5-4750T @2.90 GHz	
Physical memory (RAM)	8.0 GB	
Storage	1 TB	
Software, API(s), simulation tools		
Operating system	Microsoft Windows 10 Home	
JDK	19	
JADE	4.6.0	
JADE-LEAP	4.1.1	
Java J2ME	2.5.2_01 for CLDC	
AgentWorkbench	2.3.0	
Communication specification		
ZTE 5G wireless router	Download speed up to 150 Mbps/upload speed 50 Mbps	
Communication protocols	HTTP, RMI	

### SMDPA UML diagram design

**Figure 5:**
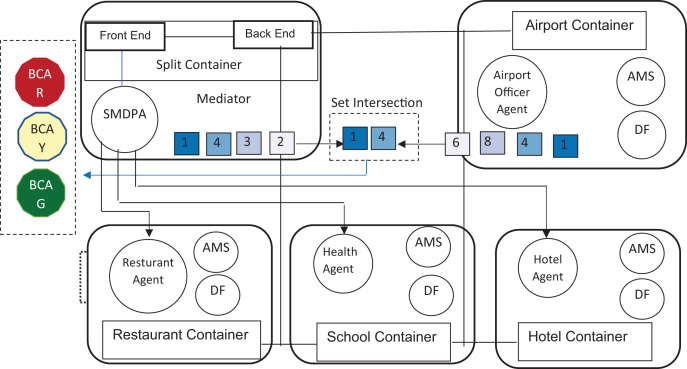
SMDPA approach execution in run time environment.

In this experiment, JADE-LEAP is executed in a split execution mode. The Jade container, as shown in Fig. 5, is split into a backend that runs on a local host and a frontend that runs on a mobile device. Such split of execution suits wireless devices that demand resource constrained (LEAP User Guide, 2003). In this research, the proposed solution was designed using five JADE containers, as shown in Fig. 5. Besides the split container described above, four additional JADE containers were built to model an airport, a restaurant, a school, and hotel facilities.

An external agent manages each container. For instance, an airport officer agent represents an immigration officer at an airport and operates the airport container. Likewise, the hotel agent manages the hotel container while the restaurant agent manages the restaurant container, and so does the school agent to the school container.

**Figure 6:**
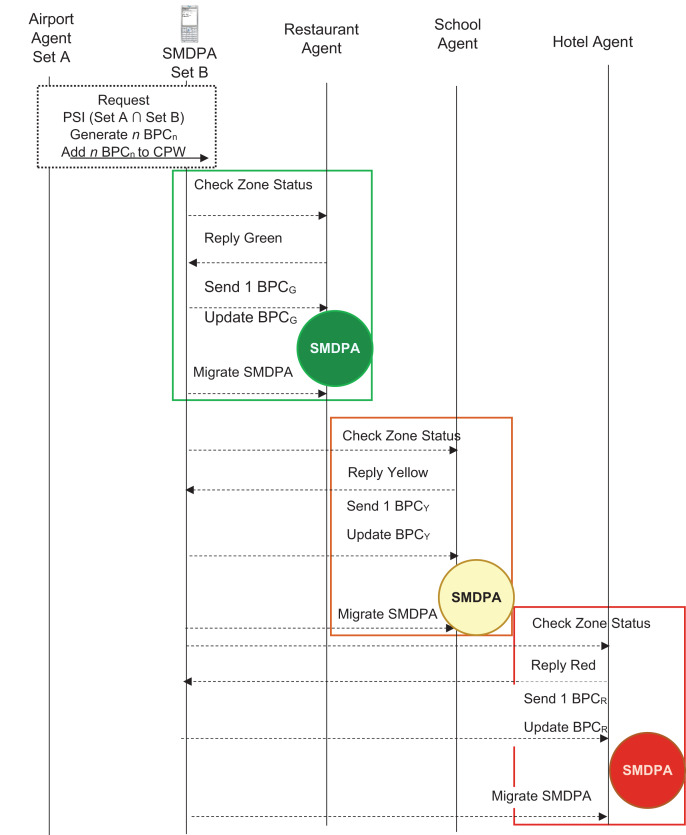
SMDPA UML sequence diagram.

Figure 6 shows a model interaction among the entities involved in the secure mobile digital passport agent (SMDPA) protocol. The process goes as follows. First, a passenger arrives at an airport and requests a border officer to digitally assess his digital health passport. Next, the officer performed a cross-border joint private set intersection (PSI) interaction with the passenger. Then, based on the intersection described above in Example 2, n BPCs are generated and deposited into the travel passenger CPW. Finally, the passenger uses one BPC_G_ to be granted safe entry.

Before interacting with the border immigration officer, a function might be triggered to evaporate data that does not comply with the privacy protection acts. The process is conducted through the location attributes that check the IP address of the destination, and that decide what data are needed to be evaporated before performing a joint privacy set intersection with the border officer. The travel passenger, afterward, moves freely. However, he might be restricted from visiting certain zones, or being granted a few visits to others. This depends on the PSI result of the first interaction with the border officer. For example, Fig. 6 demonstrates that if a traveler wants to visit a green zone area, say a restaurant, a request is sent to the restaurant agent. The restaurant agent demands a BPC_G_, and the passenger checks his coin passing wallet (CPW) account and deposits one BPC_G_. The restaurant agent then permits the passenger to enter the restaurant.

In another scenario, as shown in Fig. 6, the passenger wishes to enter a school and finds out it is modeled as a yellow zone area. The passenger sends a request to access the school campus. The school agent requests the passenger to deposit BPC_Y_ and then permits the passenger to access the school campus. In a third scenario, the passenger wishes to stay at a hotel. He sends a request and finds out that the hotel is modeled as a red zone area. He sends a bid and is asked to deposit BPC_R_, which he deposits, and is granted access. Note that, as described above, the number of issued BPCs and their levels is predicated largely upon both the passenger’s personal and health information, on the one hand, and the visited countries’ rules and restrictions, on the other hand, and all interactions are expected in a secure private manner.

#### Prototype of SMDPA solution

As a decentralized environment, the proposed scheme was prototyped using the Java agent development framework (JADE) agent framework (Bellifemine, Caire & Greenwood, 2007). It relied on several add-on libraries that each had its purpose. For example, the lightweight extensible agent platform (or LEAP) was used to modify the JADE kernel to support the run time environment for developing the JADE app for mobile devices with limited resources. JADE-Leaps splits the execution environment into two parts: a frontend that runs on the mobile and a backend that acts as a mediator. As shown in Fig. 5, the researcher created five containers, and implemented five agents, using Java classes that each manage the message’s communication with SMDPA.

In addition, the investigator used two array lists populated with integers to simulate set intersections between the SMDPA and the airport officer agent. He also implemented JADE behaviors to manage the messages exchanged among agents. Finally, he used another add-on library, Agent.Workbench (Formely Agent.GUI) (Derksen, Branki & Unland, 2011) to simulate and measure the developed prototype performance. Table 8 summarizes the computing environment the researcher used to implement and deploy the solution.

## Results and discussion

### Results

#### SMDPA Prototype evaluation using JADE and Agent.Workbench

**Figure 7:**
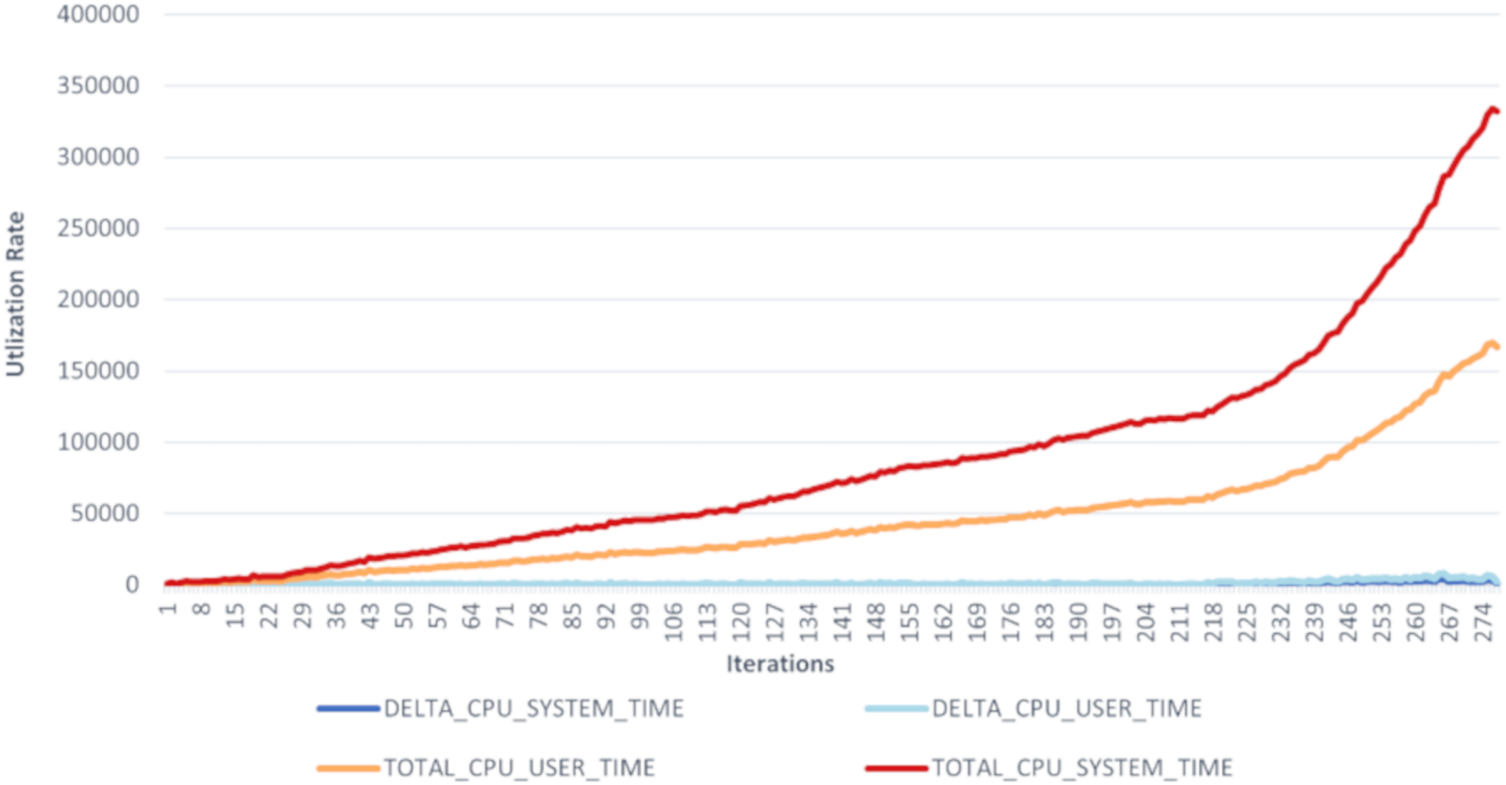
CPU load’s performance for SMDPA and the airportagent interaction.

**Figure 8:**
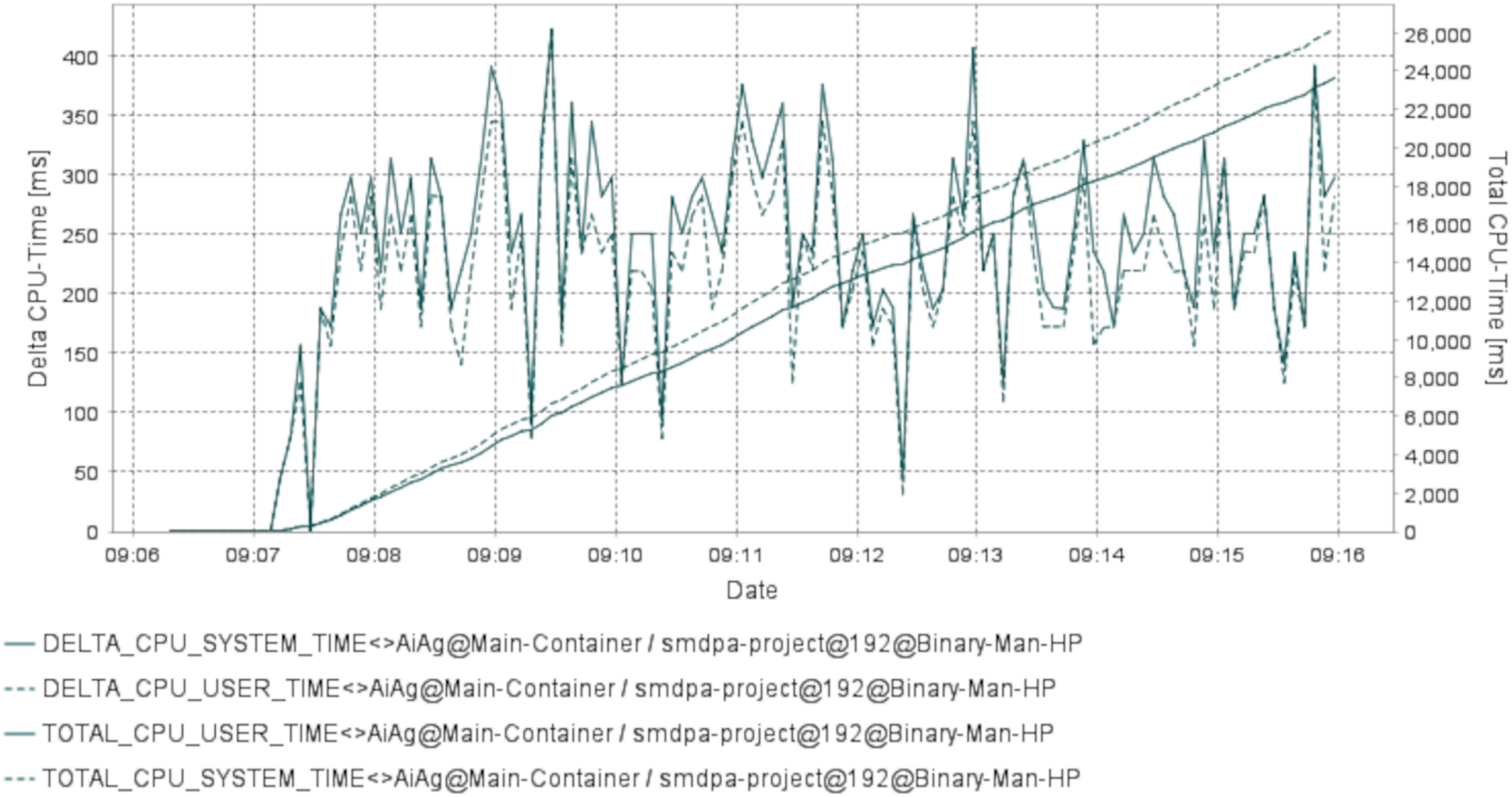
CPU load time for SMDP and the airportagent.

This research evaluated the proposed scheme’s integrated architecture prototype using Agent.Workbench (Derksen, Branki & Unland, 2011). The CPU usage is analyzed to track the agents’ CPU load on the machines. This should account for CPU resource consumption and help enhance interaction and intersection algorithms. Figures 7 and 8 show a performance chart for monitoring the experiment’s performance metrics. It measures the CPU Load’s performance during the interaction of the set between the AirportAgent and SMDPA. The performance metrics parameters are delta CPU time in milliseconds for the user, delta CPU time in milliseconds for the system, and the total CPU time for the user and total CPU time for the system. The idea is to track and observe the ways in which the proposed approach consumes CPU based on the set intersection.

The “Agent.Workbench” tool generated two hundred seventy-eight samples. The presented chart illustrates a slow increase in the CPU load during the interaction between the Airport Agent and the SMDPA. Hence, agents’ average CPU usage is lower than the device’s total CPU load.

Nevertheless, CPU user time refers to the processor’s time to execute agents’ code, such as intersection, messaging, migration, and code libraries. The CPU system time refers to the execution time for running code in the operating system kernel. Hence, the total CPU time combines the agent action CPU time, and the kernel system calls time. Likewise, CPU delta time represents CPU times spent during intervals. Note that the sampling interval in our experiment was 0.5 s.

To evaluate the efficiency of SMDPA, we created a Java scheduler class to schedule and run the system agents and used “Agent.Workbench” thread monitor to collect CPU load data at an interval of 0.5 s. The thread monitor collects performance data for the agent communication language (ACL) messages during the interaction between SMDPA and airport agent. The performance metrics parameters are delta CPU time in milliseconds for the user, delta CPU time in milliseconds for the system, and the total CPU time for the user and total CPU time for the system. The idea is to track and observe how the proposed approach consumes CPU based on the set intersection. The “Agent.Workbench” tool generated two hundred seventy-eight samples. The datasets recorded the following metrics: the Date, DELTA_CPU_SYSTEM_TIME, DELTA_CPU_USER_TIME, TOTAL_CPU_USER_TIME, TOTAL_CPU_SYSTEM_TIME.

The collected datasets are recorded into a file as comma separated values (CSV) or comma delimited. Then cleaned and transformed for analysis and visualization. The data symbolize the private set intersection between SMDPA and the airport agent.

Figure 7 shows the utilization rate, which is the percentage of CPU utilization or CPU total work for the set intersection and ACL exchange messaging or message interaction between Agent SMDPA and the airport agent. The figure also shows that the experiments involved 279 iterations (279 runs) which mean 279 set intersection and ACL messaging between the airport agent and different version of SMDPA. The figure shows during the execution or running of the experiment, the line graph for the percentage of CPU utilization when executing at the system level (kernel) grows exponentially compared to the percentage of CPU utilization at the application or user level. The CPU load is monitored to estimate and maintain application and system consistent performance to avoid system failures. This could indicate that the airport agent is recommended to run from a cloud environment relying on multiple hosts to avoid any causes for high CPU utilization.

Figure 8 shows the same data presented in Fig. 7 in different views. The date in the figure represents the point of time for capturing the CPU delta and the total time in milliseconds for the user and the system during the interaction between SMDPA and the airport agent or “AiAg”. Similarly to Fig. 7, the chart in Fig. 8 shows that CPU utilization at the user and system level increase almost linearly, and the gap between the two lines keeps increasing exponentially.

The main result is (i) creating a paradigm that integrates PSI-cardinality (PSI-CA) and agent-based solutions for an innovative digital passport. Then, (ii) developing a prototype implementation for the smart digital passport using JADE. Finally, (ii) measure the performance of SMDPA using “Agent.Workbench” and compare the result with the result of active data bundles using secure multi-party computation (ADB-SMC). The result showed the practicality of the proposed scheme since measuring multi-agent systems (MAS) is a challenge and requires agent infrastructure.

We observe that using PSI-cardinality (PSI-CA) can ensure higher privacy and performance, lower overhead, and better flexibility compared to the similar ADB-SMC that was modeled for a similar problem. Furthermore, SMDPA does not require continuous cryptographic services, as passengers can access restricted zones depending on the number and type of generated BPCs. Hence, there is no need to reveal identities, which should ensure higher security than ADB-SMC.

The utilization system time for the CPU grows exponentially compared to the percentage utilized by the agent application. Thus, using a cloud-based environment for SMDPA and the airport agent intersection and messaging might be a good solution and will be investigated in future work.

We compared the performance measurements of the proposed scheme SMDPA with the one presented by Sarhan & Carr (2017), the so-called ADB-SMC. We noted the followings:

1. Both SMDPA and ADB-SMC integrate agent-based solutions with PKI-based encryption solutions. However, the expected overhead in SMDPA is lower because its privacy policy encapsulates its data with a single layer of encryption compared with ADB-SMC.
2. SMDPA does not reveal data since it relies on PSI-CA, and ADB-SMC relies on attribute-based encryption (ABE), which allows the encryptor to decide who can decrypt the data based on attributes. Hence, SMDPA provides higher anonymity since it relies on intersection size rather than revealing or decrypting the data.
3. To measure the MAS effect on a host computer system, we measured CPU utilization data in SMDPA using “Agent.Workbench” since CPU resources can be variable and unpredictable. We observe the following:

The expected overhead in the proposed scheme is less than the one presented by ADB-SMC. This is due to the following reasons: (1) Although both methods are based on multiparty computations, the cryptographic service in the proposed scheme uses a single-layer PKI system, while the one proposed by Sarhan & Carr (2017) uses multiple layers encryption systems. (2) SMDPA performs PSI-CA only once, and then the generated BPCs control its interaction with other parties, while ADB-SMC, if employed to act similarly to SMDPA, would need to decrypt its data continuously or every time interacts with a party, so it is being observed that overall, SMDPA has lower overhead comparing to ADB-SMC.

**Figure 9:**
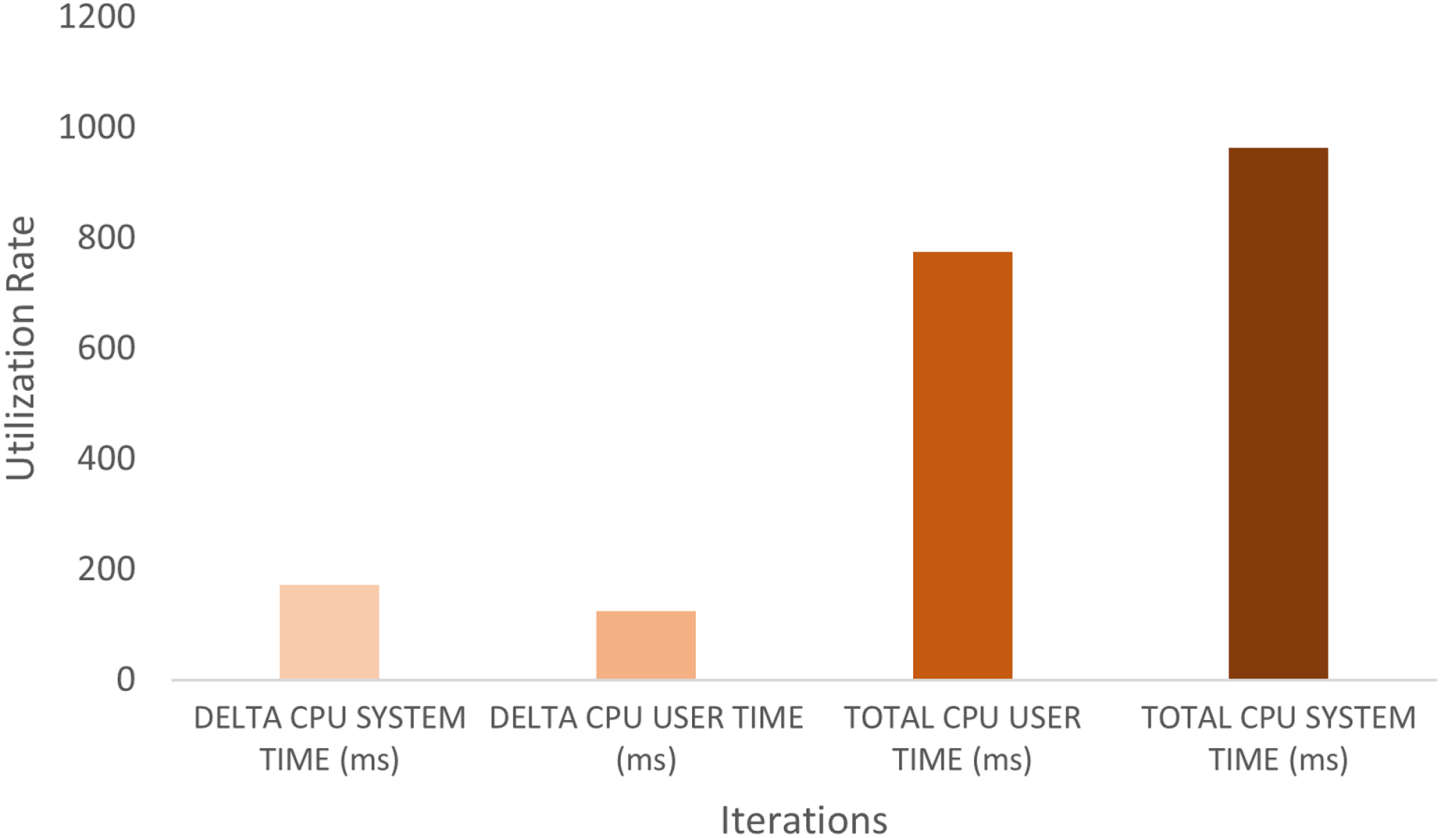
SMDPA CPU load average time.

Figure 9 shows the average CPU load for ten iterations(runs) of SMDPA. To compare the performance between SMDPA and ADB-SMC, compared with ADB-SMC (Sarhan & Carr, 2017), SMDPA has a lower average CPU load which is approximately 775 milliseconds (ms) compared to about 900 ms for ADB-SMC. Thus, SMDPA performs better than ADB-SMC.

#### SMDPA algorithms evaluations

**Figure 10:**
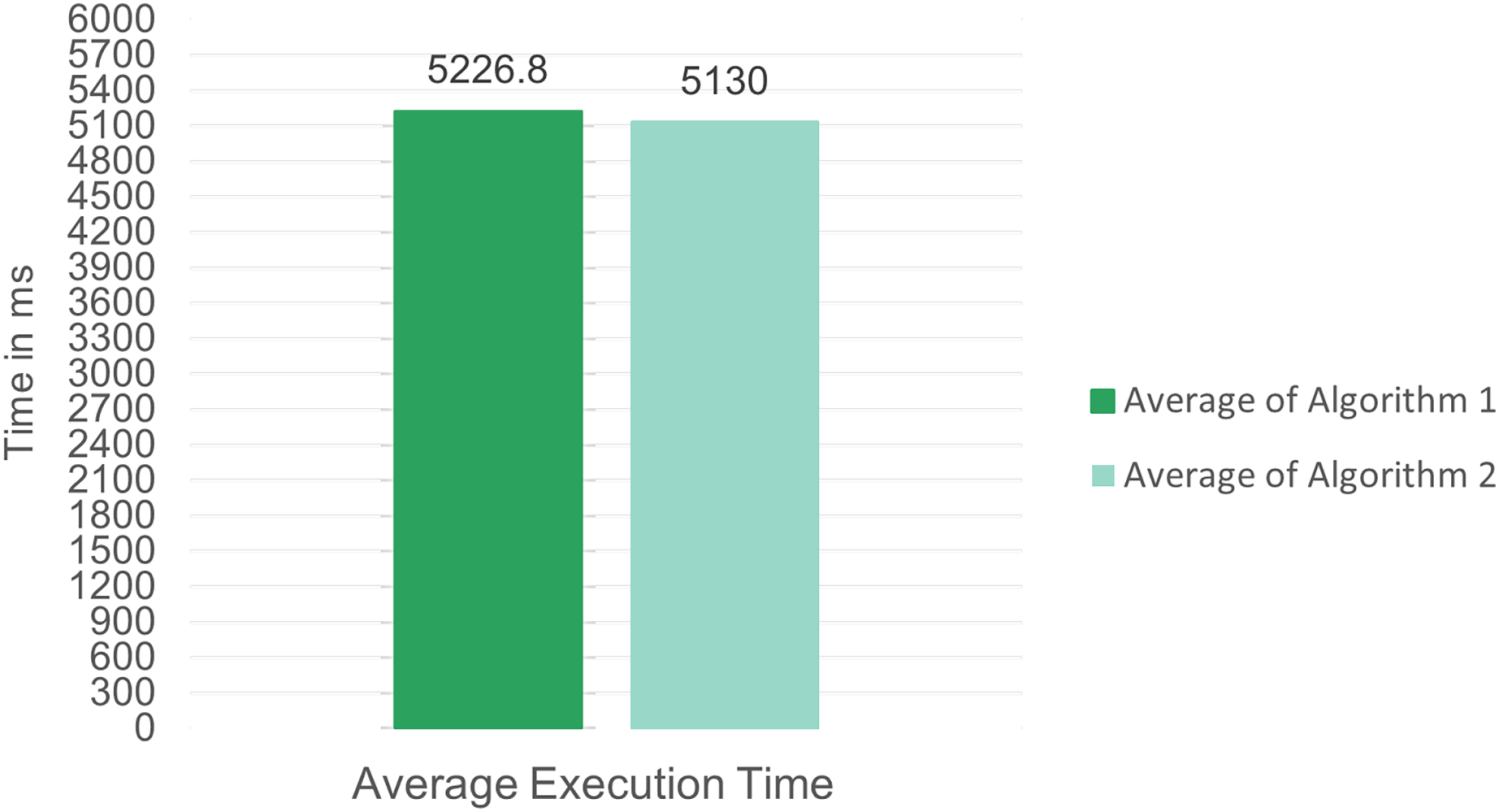
SMDPA algorithms average time.

The investigator described two algorithms for SMDPA communication and interaction with other agents in the architecture and design section. In this section, the performance of these algorithms is measured. The two algorithms are implemented using Java, and precisely measure the elapsed time for code execution using Java.System.nanoTime(). System.currentTimeMillis(). A Java random number generation function was used to model the stream generation of Bit Passing Coin (BPC) and used an ArrayList object to model CPW, so storing the generated stream of BPC. Three loops were used to create three BPC levels and measure their elapsed time. The researcher generated 250 instances for each of Algorithm 1 and Algorithm 2. Figure 10 indicates the enhanced SMDPA Algorithm 2 has a better average execution time than Algorithm 1.

## Discussion

### Simulation limitation

The result shows the viability and practicality of the proposed approach; however, the researcher simulated the private set intersection (PSI) protocol using a set of integers on the grounds that he assumes data can be encoded as integers. It falls outside the scope of this work to extend any PSI protocol, as the main purpose of this article is to highlight the practicality of agent-based solutions in modeling crises. The researcher believes that the most suited PSI protocol for this work should be a one-way PSI in which interaction is performed at the SMDPA.

The emulator used is to prove the concept of the proposed solution. As for future inquiries, the researcher plans to use smart mobile device-based android. The split execution mode used to simulate the proposed work could affect the result in contrast to the stand-alone execution mode, where a complete container could be executed on the device execution mode. The investigator used the split execution mode as recommended by LEAP User Guide (2003) as the most effective when running JADE-LEAP on a personal CLDC device where mobility features are needed. This research focused on measuring the performance overhead of SMDPA and the airport agent or first agent to interact with SMDPA, asserting that the highest overhead time should occur during the set intersection process.

## Conclusions

This article studied the problem of sharing digital health passports securely. It presented the following contributions. First, it systematically reviewed thirty-six crisis-based platforms and found that most apps lack proper privacy protocol settings and are vulnerable to several attacks. Next, it designed a secure mobile digital passport agent (SMDPA) decentralized protocol. The proposed protocol addresses the common issues seen in many typical crisis-based mobile applications, such as data leakage, surveillance, security, interoperability, and performance. Finally, a sample prototype is developed, and an experimental evaluation of the proposed protocol is performed to prove the proposed work’s concept. Our observation found the results acceptable since SMDPA average CPU load performed better with 775 milliseconds (ms) than approximately 900 ms recorded by the scheme called active data bundles using secure multi-party computation (ADB-SMC).

For future work, the researcher plans (1) to deploy the proposed work on a real smart mobile app; (ii) to try different PSI settings and filters and find the best that can suit the purpose of his work; (iii) to Integrate the proposed solution with Blockchain, and study saving SMDPA as a deserialized copy in the Blockchain; and (iv) to modify the proposed protocol by using PSI-CA version that decides if the intersection size is larger than a pre-specified threshold L and to compute some function to identify the private data that need to be evaporated.

## Supplemental Information

10.7717/peerj-cs.1458/supp-1 Supplemental Information 1 The execution time results for Algorithm 1 and Algorithm 2 execution time results. The following 250 data iterations represent the execution time results generated from running Algorithm 1 and Algorithm 2.

10.7717/peerj-cs.1458/supp-2 Supplemental Information 2 SMDPA and the Airport agent Datasets. The following data iterations were generated using “Agent.Workbench” and Jade agent for CPU load during the set intersection between SMDPA and the airport agent.

10.7717/peerj-cs.1458/supp-3 Supplemental Information 3 Code for running SMDPA Simulation.

10.7717/peerj-cs.1458/supp-4 Supplemental Information 4 Code for running SMDPA Algorithm 1.

10.7717/peerj-cs.1458/supp-5 Supplemental Information 5 Code for running SMDPA Algorithm 2.

## Additional Information and Declarations

### Data Availability

The following information was supplied regarding data availability: The code for running the simulation and the generated datasets used to assess the performance of the proposed scheme are available in the Supplemental Files.
